# Abdominal wall endometrioma after Cesarean section: a case series

**DOI:** 10.1093/jscr/rjae295

**Published:** 2024-05-07

**Authors:** Craig Biegel, Sandra Kumwong, Masaru Fujimoto, Sohayla Rostami, Aneesh Penukonda, Delcasse Joseph, Dmitriy Kim

**Affiliations:** Department of Surgery, St. John’s Episcopal Hospital, 327 Beach 19th Street, Far Rockaway, NY 11691, United States; Touro College of Osteopathic Medicine of Touro University – Harlem, 230 West 125th Street, New York, NY 10027, United States; Department of Surgery, St. John’s Episcopal Hospital, 327 Beach 19th Street, Far Rockaway, NY 11691, United States; Department of Surgery, St. John’s Episcopal Hospital, 327 Beach 19th Street, Far Rockaway, NY 11691, United States; Department of Surgery, St. John’s Episcopal Hospital, 327 Beach 19th Street, Far Rockaway, NY 11691, United States; Department of Surgery, St. John’s Episcopal Hospital, 327 Beach 19th Street, Far Rockaway, NY 11691, United States; Department of Surgery, St. John’s Episcopal Hospital, 327 Beach 19th Street, Far Rockaway, NY 11691, United States

**Keywords:** abdominal wall mass, Cesarean section, endometrioma, excision

## Abstract

Abdominal wall endometrioma (AWE) results from endometrial-like tissue implants in the abdominal wall after uterine surgery. While the diagnosis can be challenging, an abdominal mass at the site of a previous incision accompanied by cyclical pain and enlargement correlating with menstruation is highly suspicious. Excision is indicated for symptomatic relief as well as the probability of malignant transformation. Because signs and symptoms are similar to other soft tissue lesions, general surgeons are sought out for excision and thus encounter the majority of AWE cases. Here, we present two patients of similar age who both presented to our hospital within one month, each found to have an endometrioma at the site of a Pfannenstiel scar after Cesarean section, and were managed operatively.

## Introduction

Endometriosis is a benign condition defined by the presence of endometrial-like tissue in an extra-uterine region [1]. Ectopic endometrial tissue can be found in the lungs, skin, gastrointestinal tract, and abdominal wall, among other viscera [1]. Although the etiology of endometriosis remains in question, it is thought to occur from retrograde flow during menstruation [2]. Abdominal wall endometrioma (AWE) is a fairly uncommon occurrence noted in ~2% of cases and usually results from endometrial seeding of the abdominal wall after open or laparoscopic uterine surgery [3]. The incidence of endometriosis specifically after Cesarean section has been estimated at 0.03%–0.45% [4]. Signs and symptoms are similar to those experienced during menstruation and include the sensation of an enlarging mass accompanied by localized pain that corresponds to menstruation [5]. AWE may also be an incidental finding following Cesarean section with onset of symptoms ranging from months to 17.5 years with an average of 55.2 months [6]. This long range of time prior to determination of AWE as the diagnosis has been attributed to designating this pathology as other masses like desmoid tumors, suture granulomas, abscesses, hematomas, sarcomas, lipomas, metastatic lesions, or hernias [6, 7]. While excision is usually performed for symptomatic relief, it also decreases the risk of malignant transformation as AWE confers a risk of malignancy in 0.3–1% of cases with clear cell carcinoma being the most common form [8]. Although AWE may be thought of as a primary gynecologic issue, it is usually discovered by general surgeons, and so, general surgeons should be familiar with its presentation. This report not only aims to discuss two cases of AWE, one repaired with mesh and the other primarily, but also emphasizes the importance of maintaining a high index of suspicion for AWE in a patient who presents with an abdominal wall mass and history of uterine surgery to avoid misdiagnosis.

## Case series

### Case 1

**Figure 1:**
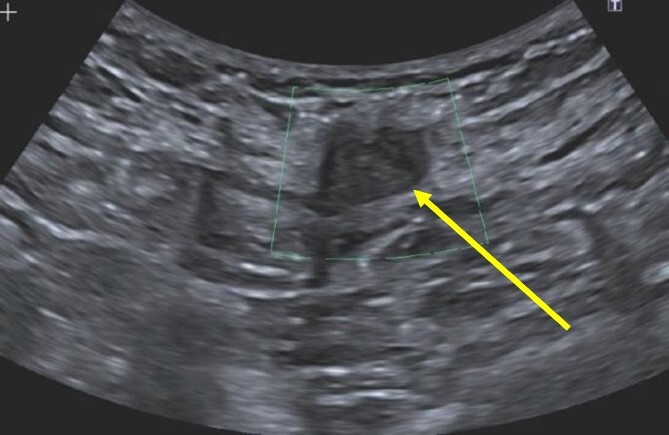
Hypoechoic, nonvascular, non-fluid-filled subcutaneous nodule (arrow), likely suture granuloma.

**Figure 2:**
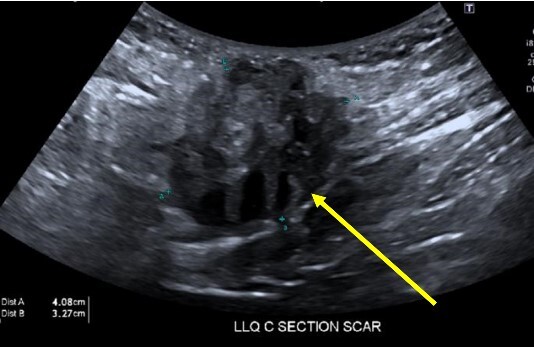
Heterogeneous, vascular nodule (arrow) measuring 4.1 × 3.3 × 4.4 cm, suggestive of endometrioma.

A 38-year-old female with past medical history of hypertension, migraine headaches, and anemia as well as past surgical history of two Cesarean sections presented to our general surgery clinic with a painful abdominal mass at the left lateral aspect of her Pfannenstiel incision. She reported that her most recent Cesarean section was 4 years ago with recurrent pain over the incision for about 2 years. Of note, 2 years prior to presentation at our clinic, she was seen by a general surgeon for similar symptoms and was found to have two subcutaneous nodules at the lateral edge of the Pfannenstiel scar. Ultrasound of the region at that time showed two hypoechoic, nonvascular, and non-fluid-filled subcutaneous nodules suggestive of suture granulomas (Fig. 1). Since then, she had experienced intermittent discomfort. More recently, each month at the time of menstruation, she stated that the mass would enlarge and become more painful. After menstruation, the mass would decrease in size and the pain would recede. On this visit, ultrasound revealed a heterogeneous vascular soft tissue mass measuring 4.1 × 3.3 × 4.4 cm, suspicious for endometrioma in the setting of her clinical history (Fig. 2).

**Figure 3:**
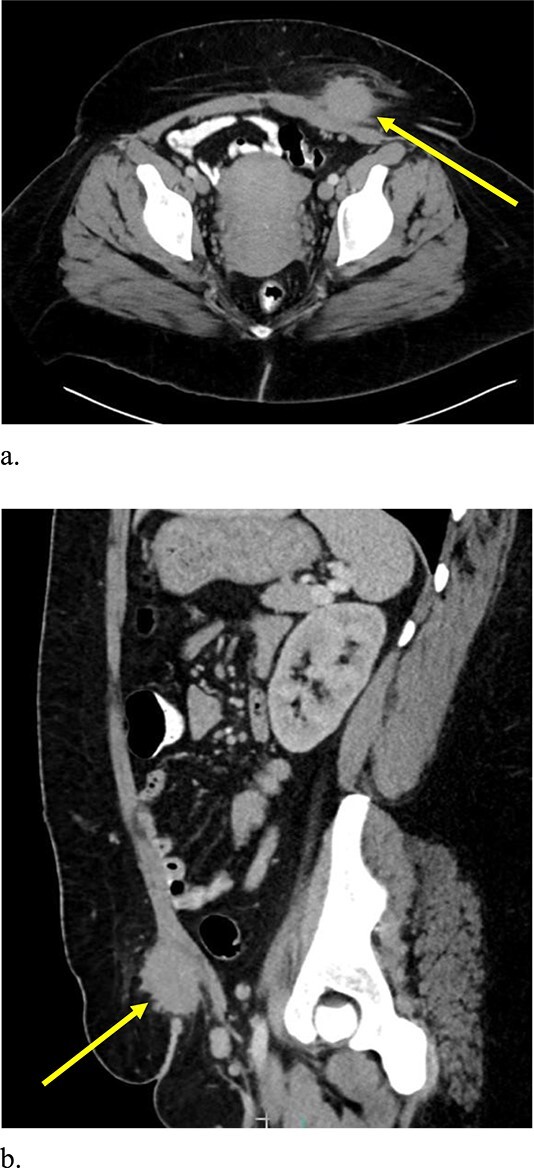
Computed tomography axial (a) and sagittal (b) views of a 4.2 × 4.1 × 3.7 cm enhancing, irregular mass on the abdominal wall (arrow) suggestive of AWE.

Computed tomography (CT) scan demonstrated a slightly larger, rounded mass with irregular, spiculated margins in the subcutaneous fat of the left ventral pelvis with fat stranding measuring 4.2 × 4.1 × 3.7 cm (Fig. 3). CT-guided biopsy of the mass was performed and revealed endometriosis. The decision was made to take the patient to the operating room for elective resection.

**Figure 4:**
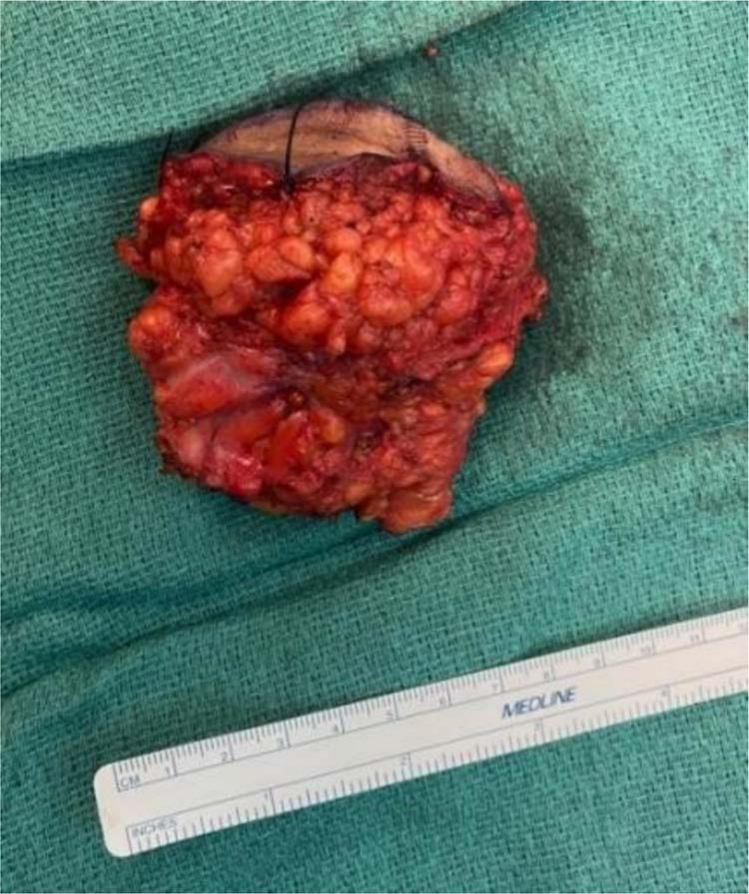
Oval yellow-tan mass measuring 7 × 6 × 4 cm.

**Figure 5:**
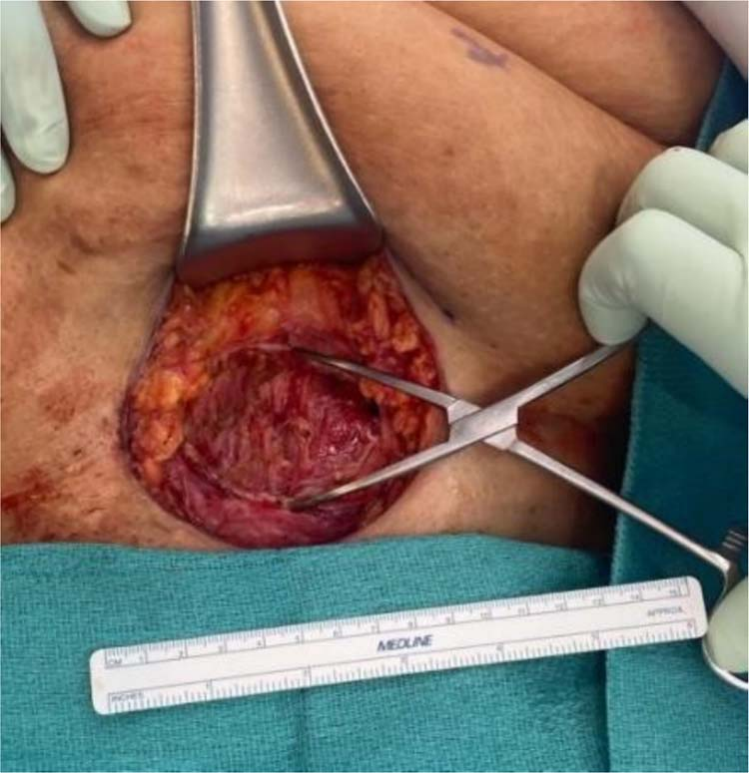
Defect in the abdominal wall after excision.

**Figure 6:**
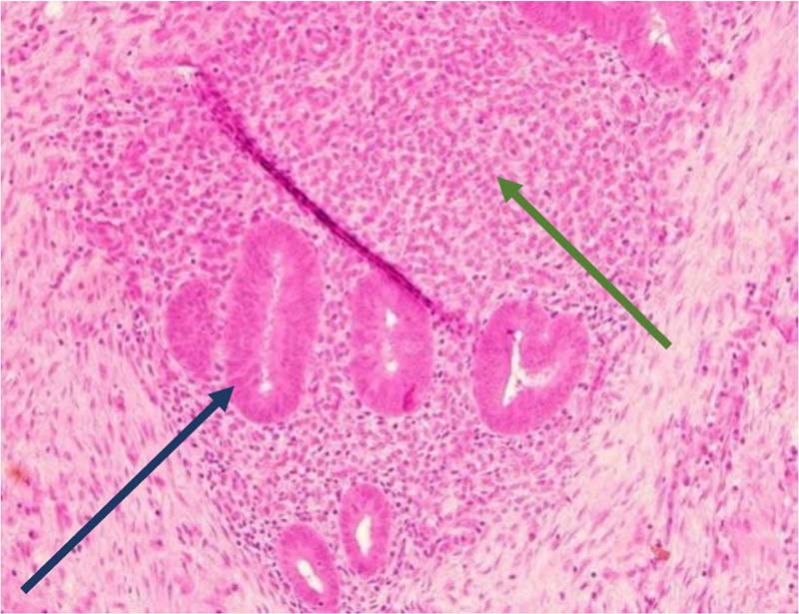
Low power view showing endometrial glands and stroma in abdominal wall tissue consistent with endometriosis (H&E).

**Figure 7:**
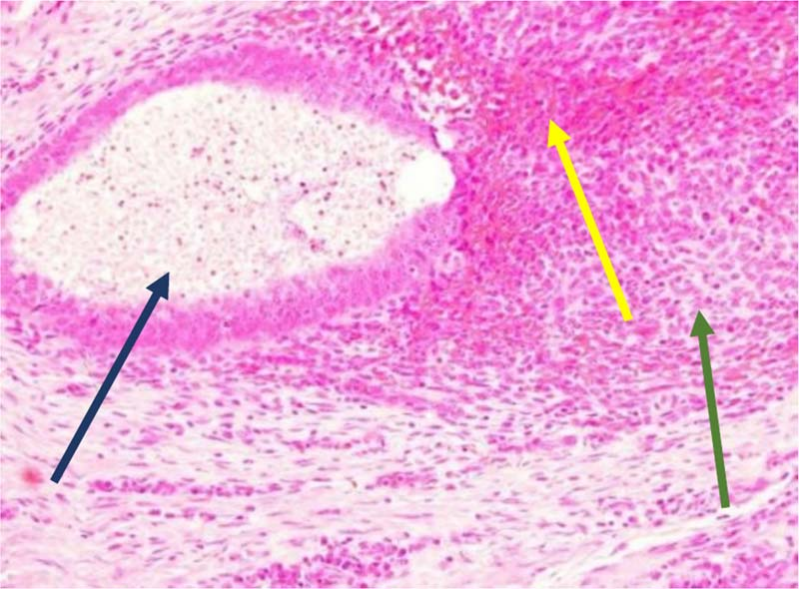
High power showing endometrial gland and stroma with hemorrhage consistent with endometriosis (H&E).

**Figure 8:**
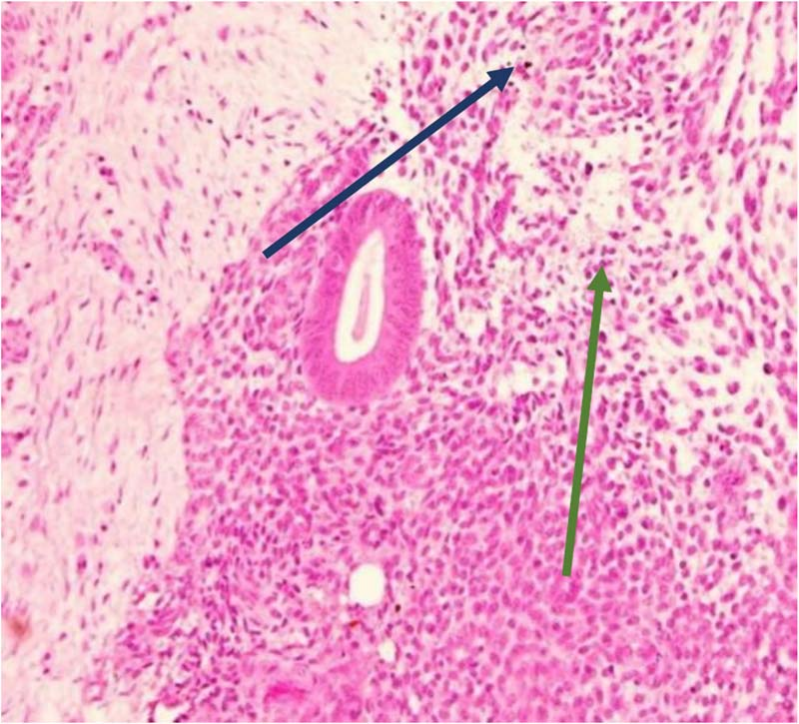
High power showing plasma cells in the stroma consistent with chronic endometritis (H&E).

The mass was located in the subcutaneous tissue of the abdominal wall just deep to the previous Pfannenstiel scar and measured 7 × 6 × 4 cm. The mass invaded the anterior rectus fascia without extension into the rectus muscle and was excised en bloc with fascia and grossly negative margins (Fig. 4). Additional margins were excised circumferentially to ensure no further remnant of endometrial cells. The defect measured approximately 12 cm and was closed with synthetic mesh (Fig. 5). Histopathology revealed endometrioma with all margins negative for endometriosis (Figs 6–8). At her postoperative visit, the patient reported complete resolution of symptoms even throughout her most recent menstrual cycle after excision.

### Case 2

**Figure 9:**
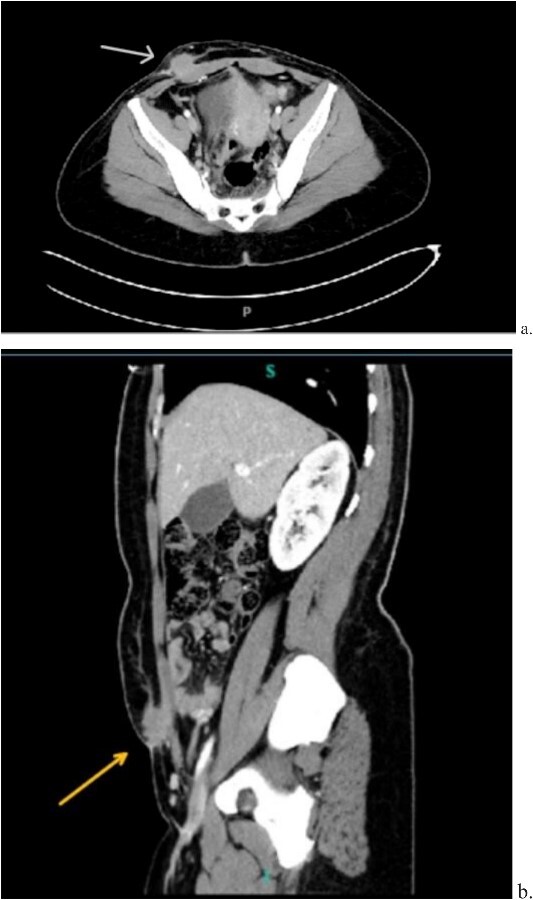
Computed tomography axial (a) and sagittal (b) views of a 2.2 × 2.0 cm enhancing mass on the abdominal wall (arrows).

A 33-year-old female with past medical history of uterine fibroids and cervical insufficiency was initially seen with peri-incisional pain at the site of her Pfannenstiel scar since an emergency Cesarean section 16 months prior for eclampsia. She reported cyclical pain since that time, worse during menstruation and associated with swelling that resolved after menstruation. The discomfort and swelling persisted for 1 week after her last menstrual period, which prompted her to present for evaluation. On examination, a palpable, exquisitely tender mass was appreciated over the right side of her Pfannenstiel incision without evidence of discharge, erythema, or ulceration. Laboratory workup proved normal, and CT imaging revealed a soft tissue abscess-like collection anterior to the rectus abdominis muscle in the lower abdomen measuring 2.2 cm without intra-abdominal involvement (Fig. 9a and b). We deemed her a candidate for operative intervention given a high suspicion for endometrioma and her symptomatology.

**Figure 10:**
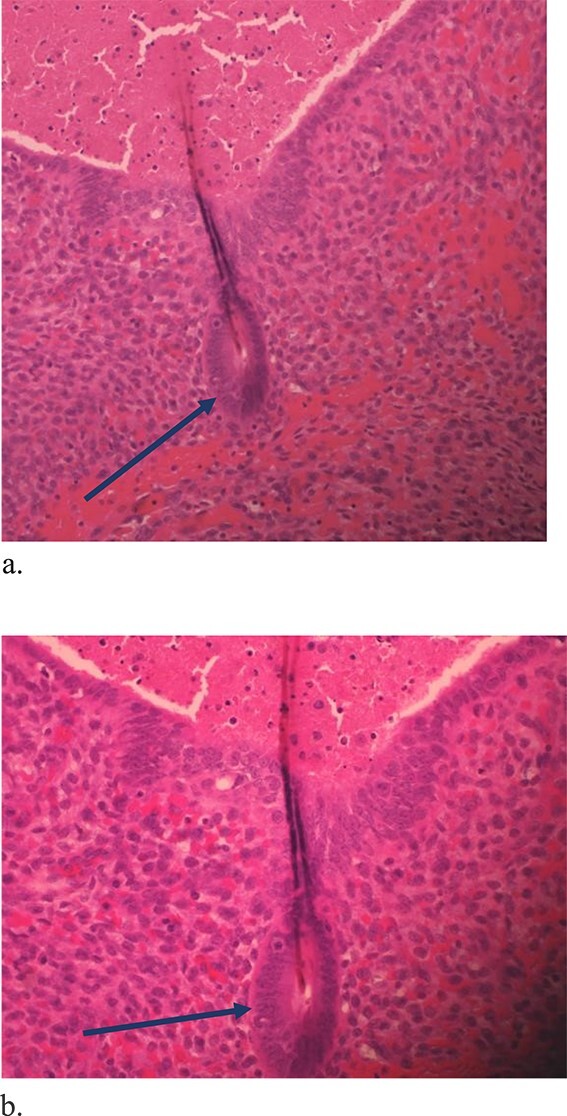
Proliferative endometrial gland (arrows) and stroma with hemorrhage in the lumen of the gland at 20× (a) and 40× (b) (H&E).

The mass was located in the subcutaneous tissue deep to the previous Pfannenstiel scar but did not invade the anterior rectus fascia. The mass was cleanly separated from the fascia and there was no invasion of the underlying muscle. During dissection, a few areas of the fascia were entered and repaired primarily. Pathology revealed endometriosis within a 4 × 3.5 × 2 cm mass with the closest margin of endometrial-like tissue measuring 2 mm from the specimen edge (Fig. 10a and b). During her postoperative visit 1 month later, the patient complained of some discomfort, yet her cyclical symptoms had resolved.

## Discussion

Endometriosis affects around 10% of women of childbearing age, of which 12% of cases are disseminated extrapelvically [9]. It can be found in a variety of organs and tissues including but not limited to the bladder, kidney, bowel, omentum, lymph nodes, lungs, pleura, hernia sacs, and the abdominal wall [10]. Abdominal wall endometriosis can occur without concurrent pelvic endometriosis [5]. The most common presentation of AWE is in the form of a mass, also known as an endometrioma, and usually follows abdominal surgery, most specifically Cesarean section. It is theorized that the abdominal wall is seeded by endometrial cells during surgery with the ultimate transformation of the growth into a mass [5].

Esquivel’s triad, which includes Cesarean section, periodic pain, and a palpable mass within a surgical scar, should raise clinical suspicion for AWE [11]. Transabdominal ultrasound is the initial imaging of choice and is often helpful in the preoperative period to rule out other common soft tissue masses [5]. On ultrasonography, AWE can appear as a cystic, solid, or mixed mass with 46.7% of AWE showing an isoechoic or hyperechoic pattern [5, 12]. The appearance of the lesions on CT imaging obtained on both patients highlighted in this report, however, is strikingly similar. Definitive diagnosis is made after tissue biopsy and surgical excision [4].

It is interesting to note that our first patient’s complaints began 2 years prior to presentation in the form of two subcutaneous nodules found in the same region as the present mass. These nodules were diagnosed as suture granulomas, a common misdiagnosis for endometrioma. The onset of suture granuloma ranges from days to years after surgery and varies in clinical presentation [13]. It can be removed surgically or it can spontaneously resolve. Ultrasonography of a suture granuloma has a characteristic hyperechoic single or double line within a hypoechoic lesion [14]. The patient’s original ultrasound at that time revealed hypoechoic nodules, though without mention of hyperechogenicity within the lesion. On clinical examination, the lesion resembled a suture granuloma. It is currently unclear whether or not these lesions were related to the endometrioma, a localized tissue reaction to newly implanted endometrium, or suture granuloma only. Regardless, incisional pain in the setting of previous uterine surgery warranted further evaluation to rule out endometriosis [15].

This condition may be more common than previously thought and accounted for in literature [1]. Prompt diagnosis is crucial to prevent delay in treatment as malignant transformation into endometrioid carcinoma, sarcoma, or clear cell adenocarcinoma has been reported [16, 17]. A review of 12 cases of AWE demonstrated transformation to clear cell adenocarcinoma with an average age of diagnosis at 45.7 years with a high associated mortality of 25% within 15 months of diagnosis [17]. Additionally, malignant transformation of AWE after Cesarean section has recently increased. Proposed thoughts for this trend are due to a general increase in the number of Cesarean sections from 7% in 1990 to 21% in the present day [16, 18]. As a result, the possibility of malignancy should be strongly considered in patients who present with Esquivel’s triad [16].

Recurrence is also an important factor to consider during resection. The recurrence rate for AWE ranges from 4.3% to 11% with 5.2 years as the mean time to recurrence after excision [16, 19]. Thus, thorough excision margins intraoperatively can confer benefits to the patient and decrease rates of recurrence: 1-cm margins have been shown to decrease the risk of recurrence. Because larger, deeper nodules that invade the rectus muscle are often associated with higher rates of recurrence, the extent of abdominal wall tissue involvement with the mass dictates repair of the defect after excision [5]. As a result, repair is determined on a case-by-case basis. In the first case, invasion of the anterior rectus sheath necessitated a wider excision with mesh closure to prevent future complications such as ventral hernia [3]. Invasion of the anterior rectus sheath was not encountered during the second case, and so, mesh repair was not indicated.

## Conclusion

Specific clues in the presentation of AWE can help prevent misdiagnosis. Resection is ultimately both diagnostic and therapeutic. Given its incidence, it is interesting that two patients with AWE presented to our institution within one month of each other. Surgical management to include mesh is dependent on fascial involvement. Additionally, it is important to pay utmost attention to a negative tissue margin not only for symptomatic relief and the prevention of recurrence, but also to decrease the risk of malignant transformation. Clinicians, especially general surgeons, should be aware of its presentation to predict the likelihood of endometrioma, and ultimately, plan for excision.
